# Effects of atmospheric-pressure non-thermal bio-compatible plasma and plasma activated nitric oxide water on cervical cancer cells

**DOI:** 10.1038/srep45781

**Published:** 2017-03-31

**Authors:** Ying Li, Min Ho Kang, Han Sup Uhm, Geon Joon Lee, Eun Ha Choi, Ihn Han

**Affiliations:** 1 Plasma Bioscience Research Center, Kwangwoon University, Seoul, 01897, Korea; 2 Department of Electrical and Biological Physics, Kwangwoon University, Seoul, 01897, Korea

## Abstract

Atmospheric-pressure non-thermal bio-compatible plasma is a partially ionized gas with electrically charged particles. Previous studies demonstrated that dielectric barrier discharge (DBD) plasma could induce apoptosis of various cancer cells, in particular demonstrating the selective cytotoxicity of cancer cells over normal cells. Therefore, DBD plasma can be considered as a potential cancer treatment method for clinical applications. We previously developed a microwave jet plasma system, producing nitric oxide called nitric oxide-plasma activated water (NO-PAW). In this study, we explored the effects of NO-PAW on a cervical cancer cell line, in comparison with DBD plasma. The cytotoxicity results showed that the treatment of HeLa cell with DBD for 4 minutes and 7 μM concentration of NO-PAW could reach almost IC_60_. For the apoptosis assay, 4 minutes treatment of DBD could induce 7% apoptotic effect, whereas 7 μM NO-PAW could induce 18% apoptotic effect. In addition, we assumed that both DBD plasma and NO-PAW could induce HeLa cell apoptosis by facilitating an accumulation of intracellular reactive oxygen and nitrogen species (RONS). Although further detail on the molecular signal pathway is still needed, DBD and NO-PAW could become promising applications for effective and safe clinical trials for cancer therapy.

Atmospheric-pressure non-thermal bio-compatible plasma has recently become a promising method for cancer therapy^1^,^2^,^3^,^4^,^5^,^6^,^7^. Dielectric barrier discharge (DBD) has been reported to destroy the intracellular structures of cancer cells such as DNA and mitochondria, causing apoptosis of the treated cells. Such destruction is mostly explained by the accumulation of intracellular reactive oxygen and nitrogen species (RONS) in cancer cells, which induce mitochondrial dysfunction and endoplasmic reticulum-stress^8^,^9^. DBD can produce variant RONS, including nitric oxide (NO), superoxide (O_2_^−^), hydrogen peroxide (H_2_O_2_), singlet oxygen (^1^O_2_), ozone (O_3_), and even hydroxyl radical (OH)^10^,^11^. These reactive species play an important role in the DBD killing effect of cancer cells; in particular, it has been demonstrated that H_2_O_2_ and NO have the most significant effect on cancer cells. Normal cells can sustain an increase in oxidative stress induced by exogenous RONS and remain below the threshold for cell death. Cancer cells have a higher basal level of RONS, and increases in oxidative stress from plasma will force them above the threshold for cell death. Consequently, this leads to a selective effect between cancer and normal cells. Even though RONS generated by plasma will be short-lived in biological components, the lipids and proteins modified by plasma-generated RONS are likely to have increased longevity and can participate in important biochemical cycles. It seems likely that some of the biochemically relevant species created in this way will be similar to species that arise naturally when the immune system creates RONS via inflammatory response to infection, tumors or wounds^12^.

Over the past few decades, NO has been one of the most important issues in life science fields because of its significant health benefits. NO also participate in various activities in living cells, as they interact with signaling molecules related to disease resistance in plants, and are associated with various circulation systems in animals. In particular, NO is important for smooth blood circulation in humans, providing various heath advantages. Indeed, NO from plasma has been applied for wound healing, showing very positive results. A NO generator based on an arc discharge mechanism has been developed and used for therapeutic purposes^13^. Na *et al*.^14^ found that NO can be very efficiently generated using a microwave torch. The NO concentration from a microwave plasma-torch can easily be controlled via the nitrogen flow rate, the mole fraction of the oxygen gas, and the microwave power. A microwave nitrogen-torch can provide the correct NO concentration for wound healing when mixing nitrogen working gas with a small mole fraction of oxygen gas. As the electrical power increases, the torch flame lengthens. The nitrogen plasma torch is very stable, and can usually operate for more than 3 hours, until all of the nitrogen in the cylindrical tank is consumed.

Plasma generated reactive oxygen species (ROS) and reactive nitrogen species (RNS) behave biochemically in the same way as the natural response of reactive species to cells and tissues when finding a biological reaction target. Plasma as a clinical application with traditional cancer therapy could lead to synergic effects for oncology treatment^15^.

In order to investigate the effects of NO-PAW on human cervical cancer cells, we studied the cell viability, pro-apoptosis effect, and intracellular ROS concentration of HeLa cells after treatment with NO-PAW. We then compared these effects with those from the DBD treatment.

## Results

### Physical characterization of Micro DBD

**Figure 1:**
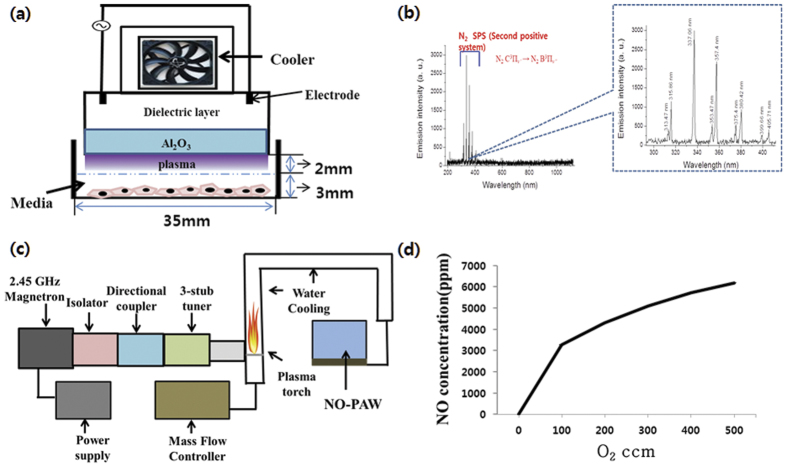
Schematic of the experimental setups. (**a**) Dielectric barrier discharge (DBD) plasma device and (**b**) measurement of optical emission spectra (OES) of the DBD device with nitrogen gas. (**c**) Schematic of the NO-PAW generator and (**d**) NO concentration under different oxygen flow rates.

Figure 1a shows a schematic of a micro DBD device, with the electrode gap of 200 μm, electrode thickness of 5 μm, dielectric layer thickness of 30 μm, and Al_2_O_3_ layer thickness of 1 μm. Plasma was discharged with a breakdown voltage of 500 V and breakdown electric current of 13 mA. Nitrogen gas was used as the working gas with a 1.5 lpm flow rate.

Figure 1b shows the optical emission spectra (OES) of DBD, indicating peaks of light ranging from 296 to 407 nm emitted from the transitions of the N_2_ second positive system (C^3^II_u_ to B^3^II_g_)^16^ and detected in plasma for N_2_ gas. These excited nitrogen species and the ambient oxygen are possibly involved in the oxidation of other molecules by their removal of electrons, which could influence many biological processes.

### Selective killing-effect induced by DBD and NO-PAW

While the selective effects of DBD are well known, those of NO-PAW are not. Thus, we attempted to investigate which scheme was able to selectively kill cancer cells and which scheme also killed normal cells.

**Figure 2:**
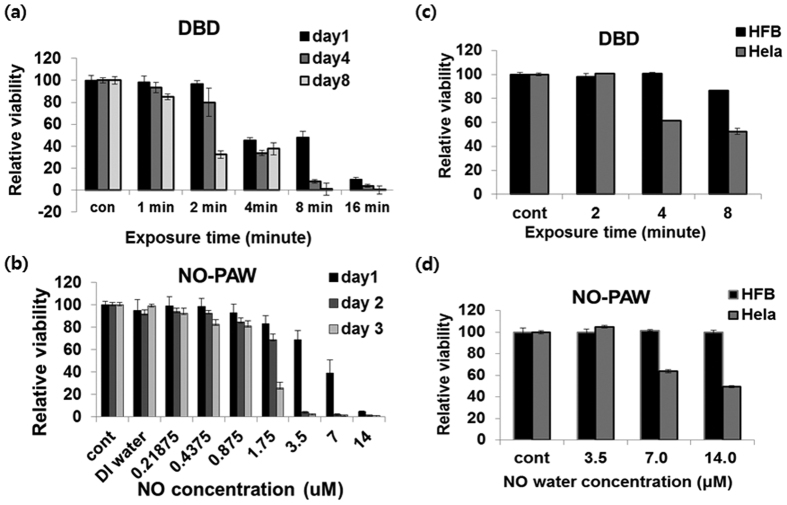
Effect of DBD and NO-PAW on cell viability. (**a**) HeLa cells were treated with DBD with different treatment times, then incubated for 1, 4, and 8 days after treatment. (**b**) HeLa cells were also treated with NO-PAW with different concentrations and incubations for 1, 2, and 3 days after treatment. (**c**) HeLa cells and HFB cells were treated with DBD for 2, 4, and 8 minutes and (**d**) NO-PAW for 3.5, 7.0 and 14 μM. MTS assay was performed 24 hours after treatment. The results are presented as the mean ± SD of three individual experiments. *P < 0.05, **P < 0.01, and ***P < 0.001.

First, we detected the cytotoxicity of DBD plasma and NO-PAW to HeLa cells. The HeLa cells were treated by DBD for various lengths of time, including 1, 2, 4, 8, and 16 minutes, and were then incubated for 1, 4, and 8 days, along with a non-treated group as the control. As shown in Fig. 2a, after 4 minutes treatment with DBD plasma and incubation for 1 day, more than 40% of the HeLa cells were killed. For the NO-PAW treatment, HeLa cells were treated with different concentrations of NO-PAW, including 0.21875, 0.4375, 0.875, 1.75, 3.5, 7.0, and 14.0 μM, and the HeLa cells were then incubated for 1, 2, and 3 days, along with a non-treated group as the control. After treatment with 7.0 μM of NO-PAW and 1 day incubation, the cell viability decreased to less than 50%. After incubation for 3 days, 90% of the cells were killed by NO-PAW, even at the lower concentration of 3.5 μM (Fig. 2b).

Secondly, we investigated the selective killing effect of DBD plasma and NO-PAW on HeLa cells and human fibroblast (HFB), serving as normal cells. The HeLa and HFB cells were exposed to DBD plasma for 2, 4, and 8 minutes (Fig. 2c), and then exposed to NO-PAW at concentrations of 3.5, 7.0, and 14.0 μM (Fig. 2d). Cell viability was detected after 24 hours incubation. As shown in Fig. 2c and d, the cytotoxicity results showed that treatment of DBD for 4 minutes and with 7 μM NO-PAW could reach an almost similar inhibitory concentration (IC_60_) to HeLa cells. On the other hand, the HFB cells showed no cytotoxic effect of NO-PAW up to 14.0 μM and DBD up to 4 minutes. DBD treatment for 8 minutes only decreased the cell viability of HFB by less than 10%. These results suggested that both DBD and NO-PAW selectively killed the cancer cells without also killing the HFB normal human cells.

### DBD and NO-PAW treatment induced apoptosis in cells

Annexin V-FITC/PI was used to confirm the pro-apoptotic effect of apoptosis in HeLa cells treated with DBD and NO-PAW. The HeLa cells were treated with DBD for three different periods of time of 2, 4, and 8 minutes, and were treated with NO-PAW at 3 different concentrations of 3.5 μM, 7.0 μM, and 14.0 μM.

**Figure 3:**
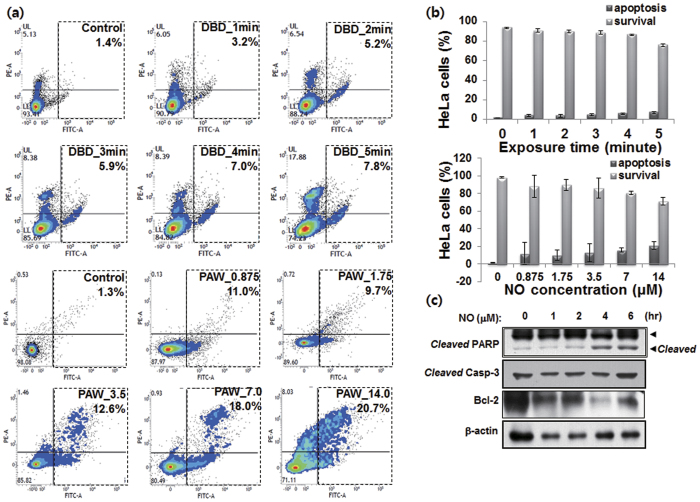
Apoptosis of HeLa cells after DBD and NO-PAW treatment. HeLa cells were treated with DBD for different periods of time (1, 2, 4, and 8 minutes), and different concentrations (0.875, 1.75, 3.5, 7.0, and 14 μM) of NO-PAW. (**a**) Representative FACS dot-plot of HeLa cells with DBD (upper) and NO-PAW (lower) were prepared with annexin V-FITC apoptosis detection kit. (**b**) Bar graph summarizing frequencies of apoptotic and survival event. Results are presented as the mean ± SD of three individual experiments. *P < 0.05, **P < 0.01, and ***P < 0.001. (**c**) Western Blot of HeLa cells was performed for 1, 2, 4, and 6 hours incubation after treatment with 7 μM NO-PAW.

Figure 3a shows that 24 h after DBD treatment (upper panel), the total percentage of apoptotic cells in the control group was 1.4%, compared with 5.2% (for 2 minutes), 7.0% (for 4 minutes), and 7.8% (for 5 minutes) in the DBD treated groups. The bottom panel of Fig. 3a shows that after 24 hours of NO-PAW treatment, the total percentage of apoptotic cells in the control group was 1.3%, compared with 12.6% (for 3.5 μM), 18.0% (for 7.0 μM), and 20.7% (for 14.0 μM) in the NO-PAW treated groups (Fig. 3b). Our findings indicate that DBD and NO-PAW could induce apoptosis in HeLa cells in a dose-dependent manner, as seen by the increasing amount of Annexin-V positive cells.

To further confirm the apoptosis induced by NO-PAW, we performed Western blot analysis on HeLa cells treated with 7.0 μM NO-PAW and incubated post-treatment for 1, 2, 4 and 6 hours. Caspase-3 cleavage plays a critical role in the apoptotic cell population, while poly (ADP-ribose) polymerase (PARP) cleavage, which is well known as a substrate for caspase-3 cleavage, is also associated with apoptosis. The Bcl-2 family is a regulation protein that can inhibit cell apoptosis. In this study, NO-PAW led to cleaved PARP by cleaved caspase-3 after 4 hours treatment of NO-PAW, and Bcl-2 started to decrease after 4 hours (Fig. 3c), suggesting that the apoptosis pathway was stimulated after 4 h of treatment of NO-PAW.

### DBD and NO-PAW treatment facilitated RONS accumulation in cells

Plasma can produce various RONS, which are important active species that can induce cell apoptosis. To determine the type of RONS involved in cell death, we detected the total ROS, specific H_2_O_2_, and NO generation inside HeLa cells after 24 h treatment.

**Figure 4:**
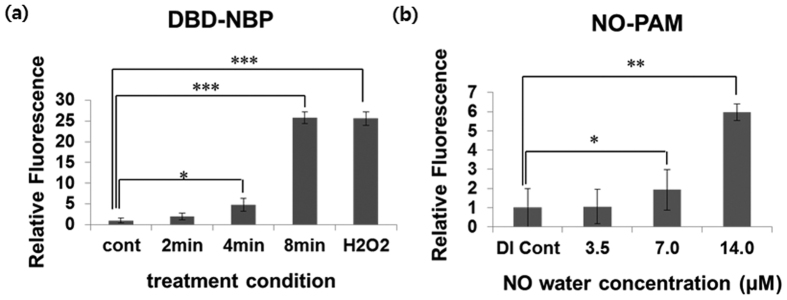
Effects of DBD and NO-PAW treatment on intracellular ROS level production. Intracellular ROS levels were measured using a fluorescent indicator H_2_DCF DA. 24 hours after treatment with DBD plasma of 2, 4, and 8 minutes and NO-PAW concentrations of 3.5, 7.0, and 14.0 μM, the HeLa cells were dyed with 20 μM H_2_DCF DA. Intracellular ROS levels increased depending on (**a**) the treatment time of DBD and (**b**) treatment concentration of NO-PAW. Results are presented as the mean ± SD of three individual experiments. *P < 0.05, **P < 0.01 and ***P < 0.001.

Initially, the HeLa cells were loaded with H_2_DCF-DA to evaluate the accumulation of intracellular ROS after treatment by DBD plasma and NO-PAW. The relative intensity of fluorescence detected by flow cytometry was proportional to the intracellular ROS level. Figure 4a showed the fluorescence intensities in groups treated with DBD were 2.0, 4.8, and 25.7, while the fluorescence intensities in groups treated with NO-PAW were 1.1, 1.9, and 6.0 (Fig. 4b). These levels were higher than those of the control groups (1.0), indicating that both DBD and NO-PAW could facilitate the accumulation of RONS in cells, which caused apoptosis to HeLa cells.

**Figure 5:**
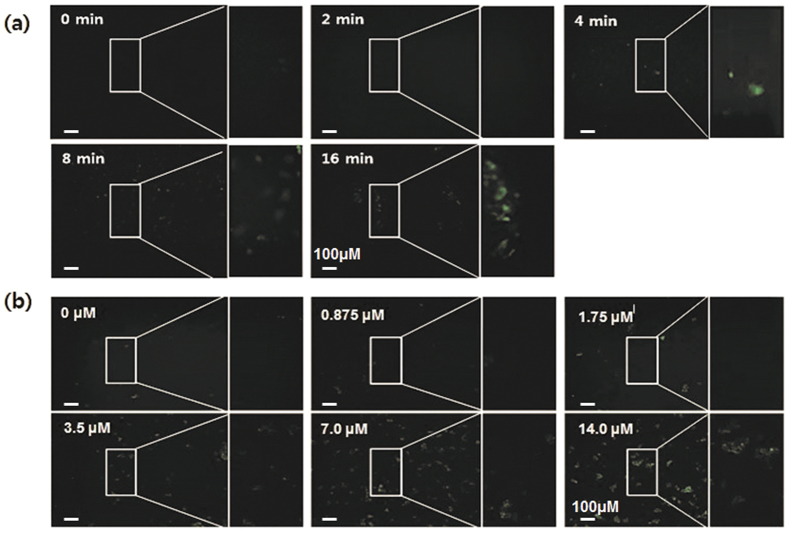
Immunofluorescence detection of HeLa cells treated with (**a**) DBD and (**b**) NO-PAW. Increasing the DBD treatment time and NO-PAW concentration resulted in stronger fluorescence inside the cells, indicating more intracellular ROS.

Both fluorescence-activated cell sorting (FACS) and fluorescence microscopy data revealed that the total intracellular ROS level increased as the DBD treatment time increased and the NO-PAW concentration increased (Fig. 5).

**Figure 6:**
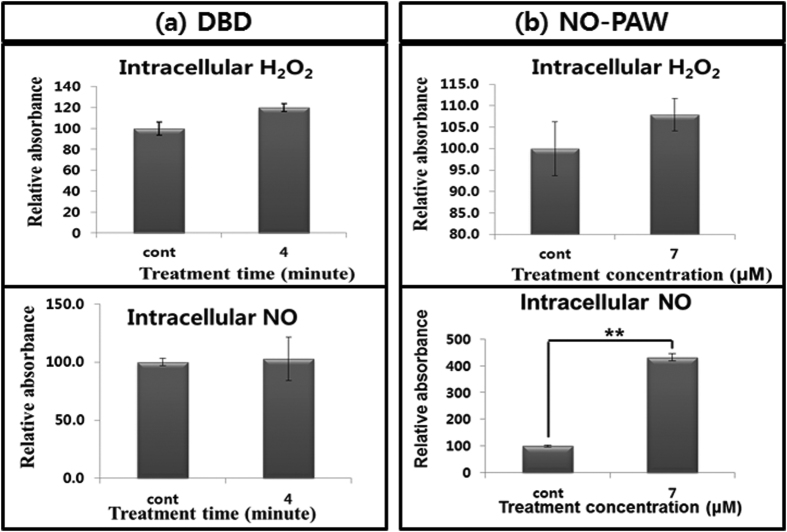
H_2_O_2_ and NO radical level of HeLa cells after DBD (**a**) and NO-PAW (**b**) treatment. Both H_2_O_2_ levels and NO radical levels increased after treatment with DBD and NO-PAW. Results are presented as the mean ± SD of three individual experiments. *P < 0.05, **P < 0.01.

In particular, NO radical and H_2_O_2_ were detected for specific intracellular RONS. As shown in Fig. 6a, 4 minutes treatment of DBD plasma induced 1.2-fold and 1.03-fold more H_2_O_2_ and NO radical, respectively, compared with the non-treated group. Also, after the treatment of 7 μM of NO-PAW to HeLa cells, the intracellular H_2_O_2_ and NO radical increased by 1.08-fold and 4.32-fold, respectively, compared to the non-treated group (Fig. 6b). Since the intracellular NO radical was significantly increased after treatment with 7 μM of NO-PAW, we consider that NO radical played the main role in inducing apoptosis in the HeLa cells with the NO-PAW treatment.

### Analysis of circular dichroism spectra of the plasma-treated HFB and HeLa cells

**Figure 7:**
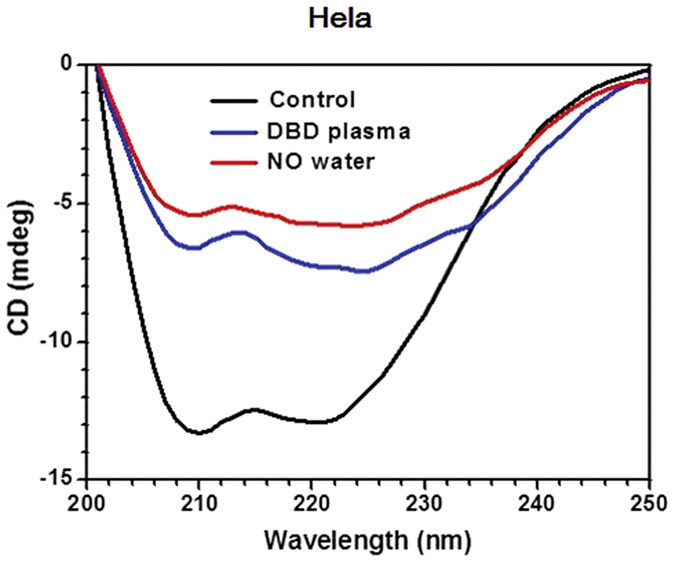
Circular dichroism spectra of HeLa cells after 4 minutes DBD plasma and 7 μM NO-PAW treatments.

Figure 7 shows the circular dichroism (CD) spectra of HeLa cells after 4 minutes DBD plasma and 7 μM NO-PAW treatments. The CD spectra of the control HeLa cells exhibited a characteristic broad band between 205 and 240 nm. The measured CD spectrum of the control HeLa cells was well fitted to the Yang model relevant to proteins. This result indicates that the chirality of HeLa cells was attributable to cellular proteins. From the secondary structure analysis, the percentage amounts of secondary structures were as follows: alpha-helix, 35.4%; beta-sheet, 4.9%; turn, 24.3%; and random coil, 35.4%. When the DBD plasma was applied to HeLa cells, the CD band intensity decreased from 13.3 to 7.4 mdeg. The CD spectroscopy indicated that a decrease in the viability of cancer cells was attributable to the destruction of cellular proteins caused by the ROS of the DBD plasma. To further study the effect of RNS on the viability of cancer cells, we measured the CD spectra of NO-PAW treated HeLa cells. Figure 7 shows that after treating the HeLa cells with NO-PAW, the CD band intensity decreased from 13.3 to 5.8 mdeg. These results indicate that the viability of cancer cells can be modified by the RNS produced by 7 μM NO-PAW.

## Discussion

The cytotoxicity assay revealed that both DBD and NO-PAW could have a killing effect on HeLa cells, while the killing effect was enhanced with increased treatment time and increased incubation time. In addition, 7 μM concentration and 2 day incubation treatment with NO-PAW showed more than 90% death of HeLa cells, compared with 60% death of HeLa cells with 4 minutes and 4 day incubation of DBD treatment. Our data revealed that, while NO-PAW had a similar killing-effect on cancer cells as that of DBD, at high concentrations, NO-PAW had a stronger killing effect than DBD.

Taking advantage of the different responses of cancer cells and normal cells to accumulated ROS, the selective killing of DBD with respect to normal cells on different cancer cells has been carried out^17^,^18^. The results showed that while DBD can selectively kill the cancer cells, it has no significant effect on normal human cells. In our study, HeLa cell and normal human fibroblast cells were treated with DBD and NO-PAW. Treatments of DBD for 4 minutes and of NO-PAW at a concentration of 7 μM both induced an IC effect on HeLa cells of around IC_60_, but showed no significant killing-effect on HFB cells. The results showed that both DBD and NO-PAW have a selective killing-effect on HeLa cells.

The ROS-mediated mechanism of selective cancer cell killing can be explained as follows. Normal cells can tolerate a certain level of exogenous ROS stress, owing to the antioxidant system in cells. Compared to normal cells, cancer cells are more vulnerable to the accumulation of ROS, due to their elevated rates of ROS production, where they promote several aspects of proliferation and metastasis in cancer cells^19^. In cancer cells, the increased ROS generation from metabolic abnormalities and oncogenic signaling can trigger a redox adaptation response, leading to the up-regulation of antioxidant capacity, and a shift of redox dynamics with high ROS generation and elimination to maintain the ROS levels below the toxic threshold. As such, cancer cells are more dependent on the antioxidant system, and are more vulnerable to further oxidative stress induced by exogenous ROS-generating agent or compounds that inhibit the antioxidant system. The further increase of ROS in cancer cells using exogenous ROS is likely to cause elevation of ROS above the threshold level, leading to cancer cell death^20^.

In order to determine the type of cell death induced in HeLa cells by DBD and NO-PAW, we used Annexin V-FITC/PI to confirm the pro-apoptosis in HeLa cells. According to the treatment by DBD and NO-PAW, the total number of apoptotic cells increased to 7% with 4 minutes of DBD and increased to 18% with 7 μM of NO-PAW. Annexin-V is a phospholipid binding protein with high affinity for phosphatidylserine (PS), which is an important component of the cell membrane. Molecules of PS normally localize at the inner surface of the cell membrane, while in apoptotic cells, they are translocated to the outer surface of the membrane, where they are readily bound with Annexin-V. DBD and NO-PAW use the same mechanism to kill the cancer cells, whereby the cell surface material is removed to expose the cell lipid bilayer. This makes it possible for extracellular RONS to easily penetrate the cells, and eventually enables the apoptosis pathway. Besides, the Western Blot results of increasing caspase-3 cleavage and PARP cleavage protein level, suggesting that the apoptosis pathway was stimulated 4 hours after treatment of NO-PAW.

In order to understand the specific changes of the membrane protein, CD analysis of HeLa cells was performed. Normally, the eukaryotic cell membrane contains many different proteins, and is vulnerable to oxidation stress. After reactive oxidative species attack, the secondary structure of protein will be changed. In particular, the β-sheet structure is important for cancer cells to maintain their cancerous property. Our results showed that after treatment with DBD and NO-PAW, the β-sheet percentage of HeLa cells decreased, indicating that the treatment reduced the cancerous character of cancer cells. However, in this case, because the α-helix percentage did not increase after treatment, we claim that DBD and NO-PAW can reduce the cancer property, but cannot yet transform cancer cells to normal cells.

In this study, we found that DBD and NO-PAW can kill cancer cells, using the same mechanism by which DBD can kill cancer cells by radical to expose the cell membrane. While NO-PAW is effective in eliminating the cancer cells leading to apoptosis. DBD can generate various radicals such as hydroxyl radical and hydrogen peroxide. This elimination can lead to the stimulation of multiple pathways of necrosis and other killing mechanisms, and thus DBD is more efficient in cytotoxicity than in apoptosis.

By comparing the apoptotic effect of DBD on cancer cells, we demonstrated that NO-PAW has the potential to selectively induce apoptosis in human cervical cancer cells, but is not toxic to normal cells. The apoptotic mechanism caused by the increase of intracellular ROS and NO radical can play the main role in this cancer cell apoptotic process. We assume that ROS from DBD and NO-PAW causes oxidative stress to cells, and initializes apoptotic process in HeLa cells. In our future work, we will investigate the apoptotic related protein and gene levels, further demonstrating that NO-PAW could be a new cancer therapy tool. We will also establish a protocol for the clinical application of NO-PAW.

## Material and Method

### Plasma device and measurement of physical properties

Figure 1 showed the structure of the non-thermal atmospheric-pressure micro DBD unit used in this study. The features of the unit have been described well in a previous study^21^. The electric power for plasma generation was provided by alternating current (AC) power supply (15.4 kHz), using a high voltage AC-AC inverter (PNP-1000, Daekwang Electric Co., Seoul, Korea). Nitrogen gas (N_2_) with a flow rate of 1.5 liters per minute (lpm) was used for plasma discharge. Current and voltage profiles during micro DBD were acquired using a magnetic pickup coil and 1000X voltage probe, respectively, with an oscilloscope (Tektronix, Beaverton, OR).

The optical emission spectra (OES) of micro DBD were measured by CCD spectrometry (HR400, Ocean Optics, Dunedin, FL). The intensity of the light emitted from the device was recorded in terms of wavelength.

### Microwave NO-PAW producer

Nitric oxide (NO) was produced using a microwave plasma torch system using 2.45 GHz microwave and 400 W power, as described in the previous study^22^. A mixture of nitrogen and oxygen gases was provided for the system for nitric oxide generation. Nitrogen gas was provided at a fixed flow rate of 10 lpm, while the oxygen-gas flow rate was provided at 100–500 standard cubic centimeters per minute (sccm), in order to produce different amounts of NO concentration, which was measured by UniGas 1000 + gas analyzer (Eurotron, Italy). NO gas reacts with oxygen to generate NO_2_^−^ and NO_3_^−^. The oxygen dissolved in deionized (DI) water needs to be eliminated in order to increase the concentration of the NO gas in the water, and decrease the dissolving time. Therefore, N_2_ gas was infused for 1 hour through a diffuser in a circular vessel (130 mm diameter, 185 mm high) containing 1 liter of DI water in order to remove the dissolved oxygen. NO gas was then injected into the container for 50 minutes (NO concentration was 5,712 ppm when the oxygen concentration increased to 400 ccm (Fig. 1d). After NO gas injection for 50 minutes, the concentration of the dissolved NO (140 μM) in the NO-PAW was measured using a Nitric Oxide assay kit (Bio Assay System, USA). For experimental use, the NO-PAW was diluted into different concentrations of 0.21875, 0.4375, 0.875, 1.75, 3.5, 7.0, and 14.0 μM.

### Cell culture

Human cervical-cancer cell line, HeLa cells, and human fibroblast cells, detroit551 were purchased from the Korean Cell Line Bank (KCLB) Korea, and cultured in Eagle’s minimal essential medium (MEM, Welgene, Kr) supplemented with 10% heat-inactivated fetal bovine serum (FBS, Gibco, NY, USA) and 1% antibiotic (penicillin/streptomycin, Gibco). The cells were grown in an incubator at 37 °C in a humidified atmosphere with 5% CO_2_. After attaining confluence, the cells were detached with 0.25% trypsin, and seeded onto a 6-well plate at the density of 2 × 10^5^ cells per well. Prior to DBD and NO-PAW treatments, the culture medium was changed to 2 ml of serum-free MEM culture medium.

### Cell Metabolic Activity Determination (MTS Assay)

MTS [3-(4,5-dimethylthiazol-2-yl)-5-(3-carboxymethoxyphemyl)-2-(4-sulfophynyl)-2H-tetrazolium] was used as a reagent to detect cell metabolic activity (viability). MTS is reduced by the cells to soluble formazan compounds by the dehydrogenase enzymes in cells that are metabolically active. HeLa cells were seeded into culture plates at a concentration of 2 × 10^5^ cells/35 mm culture dish in 2 ml of MEM media, and incubated for 24 hours prior to each experiment.

For DBD treatment, HeLa cells were exposed to DBD for 1, 2, 4, 8, and 16 minutes. For NO-PAW treatment, we added different concentrations of 0.21875, 0.4375, 0.875, 1.75, 3.5, 7.0, and 14.0 μM to the cell culture media. The HeLa cells were then incubated for 1, 4, and 8 days for the DBD group, and for 1, 2, and 3 days for the NO-PAW group. We used a control group without DBD treatment or NO-PAW treatment in each test. MTS solution was added to each plate, 4 hours before each desired amount of time. Absorbance was detected at 490 nm using a microplate reader (Biotek, VT, USA). All assays involved at least three independent sets of tests. The results are expressed as viability percentages (%). Viability (%) = (OD in sample well/ OD in control well) × 100.

### Cell apoptosis assay

To confirm the pro-apoptotic effect of plasma, cell apoptosis was measured using a fluorescence-activated cell sorting (FACS) machine (BD, USA), with an annexin V-FITC apoptosis detection kit (BD, USA). Briefly, adherent cells were collected. The cells were then suspended in 500 μL binding buffer, and stained with annexin V-FITC and propidium iodide (PI) at room temperature for 5 minutes in the dark. After removing the unbound annexin V-FITC and PI by centrifugation, the cells were resuspended in excess binding buffer.

### Intracellular ROS detection

Reactive oxygen species (ROS) plays a major role in apoptosis pathways. If cells have excessive levels of ROS, damage to the cell components generally occurs, ultimately leading to cell death. 2′7′-dichlotodihydrofluorescein diacetate (H_2_DCF DA; Invitrogen, USA) was used as a total ROS indicator. HeLa cells were seeded at a density 2 × 10^5^ cells in each 35 mm dish and incubated for 24 hours. The cells were then treated with DBD plasma for 2, 4, and 8 minutes and with NO-PAW at concentrations of 3.5, 7.0, and 14.0 μM. 24 hours after treatment, the cells were loaded with 20 μM H_2_DCFDA and incubated for 30 minutes in a dark condition. The cells were then washed twice with phosphate buffered saline (PBS) to remove the extra H_2_DCFDA, and each group of cells was then collected for detecting on flow cytometry. To confirm our results, we prepared samples of DBD plasma treated for 2, 4, 8, and 16 minutes and NO-PAW treated at concentrations of 0.875, 1.75, 3.5, 7.0, and 14.0 μM. After 24 hours, 20 μM H_2_DCFDA was loaded to each sample and incubated for 30 minutes in a dark condition. After washing twice with PBS, we observed the fluorescence intensity under a microscope (Olympus, Japan).

Additionally, since H_2_O_2_ and NO are mostly demonstrated to have the most significant effect on cancer cells, we detected the intracellular H_2_O_2_ and NO levels after treatment using the QuantiChromTM Peroxide Assay Kit and Nitric Oxide Assay Kit (BoiAssay System, CA, USA), respectively. The experiment procedures were performed following the kit instructions.

### Western Blot analysis

In order to extract protein, HeLa cells were treated with 7.0 μM of NO-PAW for 1, 2, 4, and 6 hours and the cells were then collected. For Western blot analysis, protein was separated using 4–15% sodium dodecyl sulfate-polyacrylamide gel electrophoresis (SDS-PAGE) (BIO-RAD), and was then transferred to nitrocellulose membrane (Millipore, MA, USA). After blocking with 3% bovine serum albumin (BSA), the membrane was incubated with the respective primary antibodies specific for Cleaved PARP (Cell Signaling), Cleaved Caspase-3 (Cell Signaling), Bcl-2 (Cell Signaling), and β-actin (SIGMA). Subsequently, HRP-conjugated secondary antibodies of goat anti-mouse and goat anti-rabbit (Serotec) were used to detect the corresponding primary antibodies. Immunoblot was developed using enhanced chemiluminescence.

### Measurement of circular dichroism spectra

In order to determine the effect of plasma treatment on the HeLa cells, we measured the circular dichroism spectra of HeLa cells before and after DBD plasma and NO-PAW treatment. For circular dichroism spectroscopy, HeLa cells at a density of 4 × 10^5^ cells/mL were seeded in a 35 mm cell culture dish. The seeded cells were exposed to plasma for 4 minutes and to 7 μM of NO-PAW, and further incubated for 24 hours. HeLa cell pellets were washed with PBS, and cell suspension was made in PBS for circular dichroism analysis. The circular dichroism spectra of HeLa cells were measured by circular dichroism spectrophotometry (CDS, Jasco, J-815).

### Statistical Analysis

All values are represented by the mean ± standard deviation (SD) of the indicated number of replicates. Statistical analyses of the data were performed using the Student’s t-test to establish the significance between data points, and the significant differences are based on *P* < 0.05, *P* < 0.01 and *P* < 0.001.

## Additional Information

**How to cite this article**: Li, Y. *et al*. Effects of atmospheric-pressure non-thermal bio-compatible plasma and plasma activated nitric oxide water on cervical cancer cells. *Sci. Rep.* **7**, 45781; doi: 10.1038/srep45781 (2017).

**Publisher's note:** Springer Nature remains neutral with regard to jurisdictional claims in published maps and institutional affiliations.
